# Evidence for biased agonists and antagonists at the endothelin receptors

**DOI:** 10.1016/j.lfs.2016.02.069

**Published:** 2016-08-15

**Authors:** Janet J. Maguire

**Affiliations:** Experimental Medicine and Immunotherapeutics, Level 6 ACCI, Box 110 Addenbrooke's Hospital, Cambridge CB2 0QQ, UK

**Keywords:** G protein coupled receptors, Endothelin, ET_A_, ET_B_, Biased agonism, Biased antagonism, β-Arrestin, Bosentan, Pathway selectivity, IRL1620

## Abstract

Biased ligands represent a new strategy for the development of more effective and better tolerated drugs. To date there has been a paucity of research exploring the potential of ligands that exhibit either G protein or β-arrestin pathway selectivity at the endothelin receptors. Re-analysis of data may allow researchers to determine whether there is existing evidence that the endogenous ET peptides or currently available agonists and antagonists exhibit pathway bias in a particular physiological or disease setting and this is explored in the review. An alternative to molecules that bind at the orthosteric site of the ET receptors are cell penetrating peptides that interact with a segment of an intracellular loop of the receptor to modify signalling behaviour. One such peptide IC2B has been shown to have efficacy in a model of pulmonary arterial hypertension. Finally, understanding the molecular pathways that contribute to disease is critical to determining whether biased ligands will provide clinical benefit. The role of ET_A_ signalling in ovarian cancer has been delineated in some detail and this has led to the suggestion that the development of ET_A_ G protein biased agonists or β-arrestin biased antagonists should be explored.

## Graphical abstract

Pathway bias at the ET receptors.

**Figure:**
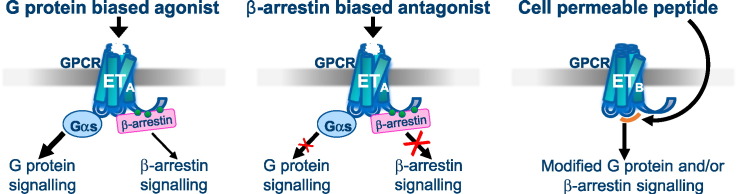

## 1 Introduction

Our understanding of how ligands interact with G protein coupled receptors is evolving, particularly the recognition that some have the ability to preferentially activate a subset of intracellular signalling cascades – so called pathway biased ligands [1]. Additionally, it is now accepted that recruitment of β-arrestin that occurs following activation of the majority of GPCRs not only results in receptor desensitisation and subsequent internalisation but may also contribute to cellular responses involved in normal physiology and disease such as cell migration and proliferation [2]. Therefore, exploiting ligand bias is likely to lead to the development of more effective and better tolerated medicines. This has so far been most clearly demonstrated for the μ opioid receptor where the agonist TRV130, a molecule that discriminates between beneficial analgesia and detrimental adverse effects such as respiratory depression and nausea, exhibited an improved therapeutic profile compared to morphine in a randomized, double-blind, placebo-controlled, crossover study in healthy volunteers [3]. Whereas bias has been considered a property of synthetic ligands it has recently been reported that for example endogenous opioids also show bias at the μ-opioid receptor [4] indicating that the presence of multiple ligands for a receptor, rather than simply representing physiological redundancy, may allow for nuanced cell specific signalling. Distinct roles for the three endogenous endothelin (ET) peptides are emerging in development and in, for example, ovarian physiology but whether pathway bias may contribute to the physiology and pathophysiology of the endogenous peptides in the ET system has not been explored. In contrast the potential for targeting the endothelin receptors with synthetic biased ligands is starting to be considered. This brief review discusses current research on biased signalling at the ET receptors and therapeutic areas of interest.

## 2 ET receptors and probe dependence

Some of the pharmacology of the endothelin receptors has over the last 20 years been described as atypical; not conforming to the basic tenets of receptor pharmacology. Particularly, this has been in differences in the behaviour of the endogenous peptides and synthetic agonists with respect to reversal by washout or blockade/reversal of responses by antagonists in in vitro studies [5], [6]. It is now apparent that for a particular receptor multiple active conformations, rather than just one, are possible and ligands can stabilize different conformations of a receptor that may activate subsets of available down-stream pathways. Therefore, some of the atypical pharmacology reported for ET receptors may be consistent with these agonists showing a degree of functional selectivity, although differences in ligand-receptor kinetics may also contribute to these observations. Additionally, because of the allosteric nature of the interaction of ligand–GPCR–intracellular protein (e.g. G protein) affinity measured in binding assays may differ from affinity measured in functional assays, specifically if different agonists stabilize particular receptor conformations then this allows the potential for orthosteric antagonists to demonstrate agonist specific functional affinities – consistent with previously reported atypical pharmacology of probe dependence [7].

## 3 Calculating ligand bias for the endogenous ET peptides and related sarafotoxin 6b at the ET_A_ receptor

**Fig. 1:**
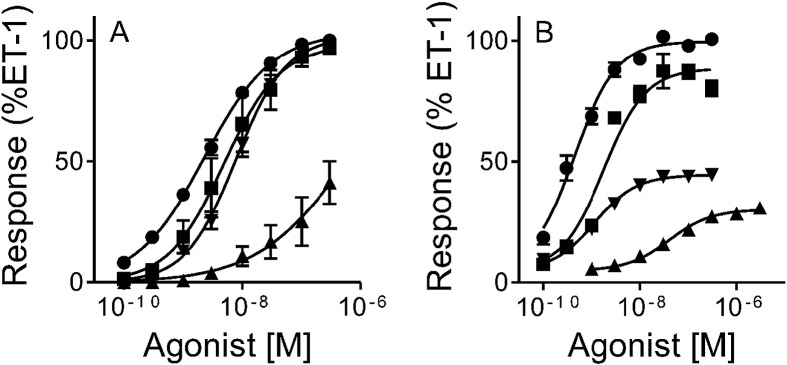
Concentration response curves to ET-1 (●), ET-2 (■), ET-3 (▲) and S6b (▼) in (A) the human endothelium-denuded saphenous vein and (B) an ET_A_-mediated β-arrestin recruitment assay. Data are expressed as a percent of the maximum response of ET-1 in each assay and data points are the mean ± s.e.m. of 3–13 experiments.

There has been at least one report in vitro that ET peptides exhibit bias at the ET_A_ receptor with ET-1 and ET-2 suggested to elicit their long lasting constrictor responses via different mechanisms that was also vascular bed dependent [8]. We have previously published data on both the potency and efficacy of endothelin peptides and sarafotoxins as constrictors of human saphenous vein [9] and in β-arrestin recruitment assays [10]. These data highlighted differences in the relative potencies and efficacies of these agonists in the ET_A_ mediated constrictor and β-arrestin recruitment assays indicative of bias. Several methods for determining pathway bias from such data have been reported including determination of transducer coefficients τ/K_A_, as described by van Westhuizen and colleagues [11]. We have applied this method to our existing data to determine whether the endogenous ET peptides and related sarafotoxin 6b (S6b) show any evidence of bias in the G protein dependent vasoconstrictor assay and G protein independent β-arrestin recruitment assay. Determining bias requires designation of a reference compound that is preferably the endogenous ligand. For the cardiovascular ET_A_ receptor the most appropriate reference ligand is ET-1. All data are expressed as a % of the maximum ET-1 response and analysed as described [10], to obtain values of log_10_(τ/KA) that are used for subsequent determination of bias factors. Fig. 1 shows that whilst ET-1, ET-2 and S6b are full agonists in the constrictor assay (the ET-3 curve is incomplete at the maximum possible bath concentration) ET-3 and S6b are both partial agonists in the β-arrestin assay.

Compared to ET-1, all agonists tested showed a 2–4 fold bias for the G protein constrictor assay compared to the β-arrestin assay (Table 1). This preliminary analysis indicated that at least modest pathway bias for endogenous ET peptides is possible, however the physiological significance of this, if any, requires more comprehensive analysis of data for ET-1, ET-2 and ET-3 in a broader range of relevant pathway specific assays.

## 4 Ligand bias at the ET_B_ receptor

There are currently no published data exploring biased agonism at the ET_B_ receptor. There are a number of ET_B_ agonists available for study including the endogenous peptide ET-3 and related sarafotoxin 6c (S6c) in addition to peptide agonists such as BQ3020 and IRL 1620. IRL-1620 is of particular significance as it is under investigation in a number of therapeutic areas with efficacy demonstrated in animal models of stroke [12] and as an adjunct for improved delivery of chemotherapy targeting solid tumours [13]. It would therefore be of interest to determine the relative effect of these agonists in a number of disease relevant pathways, with comparison to ET-3 responses to determine evidence of bias. These types of studies may highlight any differences between the agonists investigated that could be used either to further understand the signalling of importance to disease progression or to refine clinical efficacy of drugs by reducing on target detrimental effects through defined pathway activation.

## 5 Do ET receptor antagonists show pathway bias?

Of perhaps more consequence for the ET system is the possibility that antagonists exhibit pathway bias. This has been reported for the dual ET_A_/ET_B_ antagonist bosentan. In human cloned receptors bosentan exhibits a modest 20 fold selectivity for the ET_A_ receptor [14] and in human heart that expresses both receptor subtypes bosentan competes for the binding of [^125^I]ET-1 with a single affinity (K_D_: 78 nM) indicating that it does not distinguish between the native receptors in this tissue [15]. In human blood vessels that express predominantly ET_A_ receptors bosentan exhibited, as expected, 2–20 fold higher affinity than in heart with K_D_ of 32 nM in saphenous vein [16] and 3 nM in coronary artery [17]. In contrast bosentan was a much less effective antagonist than would be predicted from its binding affinity in both ET_A_ mediated vasoconstriction in human saphenous vein and coronary artery [15], in ET_B_ mediated smooth muscle contraction [14] and ET_B_ β-arrestin recruitment experiments [10] with a functional affinity of about 2 μM in all these assays. Unexpectedly, in the ET_A_ mediated β-arrestin assay bosentan was 200 fold more effective an antagonist with K_B_ of 10 nM [10] suggesting that bosentan is an ET_A_ β-arrestin biased antagonist. It is interesting to speculate that the relative effectiveness of bosentan in treatment of pulmonary arterial hypertension compared to its generally low potency as an antagonist in vitro may in part be explained by the greater antagonism of detrimental ET_A_ linked β-arrestin mediated ERK1/2 signalling [18] that could contribute to smooth muscle cell proliferation in this disease.

## 6 Alternative strategies: cell penetrating peptides as biased antagonists

Cell penetrating peptides (CPPs) are a superfamily of peptides that interact with an intracellular segment of a G protein coupled receptor and interfere with signalling [19]. Pepducins-lipidated CPPs, have been developed for over 20 GPCRs including proteinase activated (PAR1, PAR2 and PAR4) and chemokine (CXCR1, CXCR2 and CXCR4) receptors. There has been one report of a CPP, IC2B, targeting the second intracellular loop of the ET_B_ receptor which has been shown to attenuate pulmonary Akt and ERK signalling and to blunt the development of hypoxic pulmonary hypertension in a rat in vivo model [20] that is thought to contribute to disease progression. What was most interesting is that blockade of the ET_B_ receptor has previously been reported to enhance development of pulmonary arterial hypertension in rodents suggesting that in the study by Green and colleagues [20] IC2B may be selectively targeting those ET_B_ pathways contributing to muscularisation of the pulmonary arterial smooth muscle. This would leave unopposed beneficial ET_B_ receptor functions such as release of endothelial derived dilators, although this is yet to be confirmed.

## 7 Clinical potential of ET receptor biased ligands

**Fig. 2:**
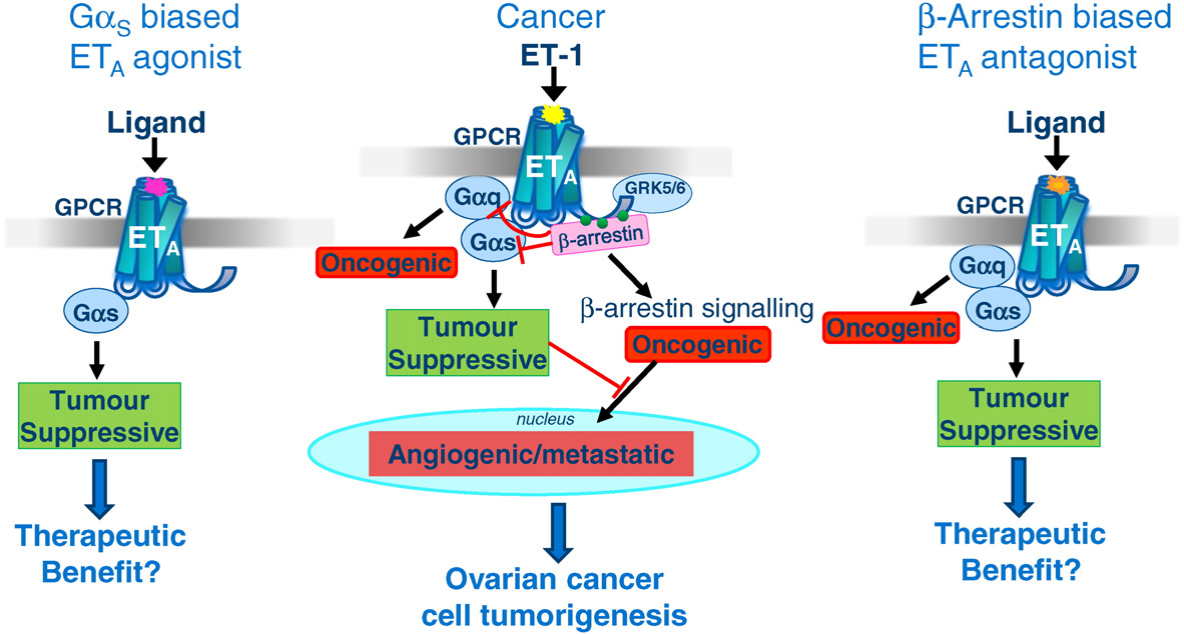
Proposed role for ET-1 activation of ET_A_ receptors in ovarian cancer (modified from [24]) and potential beneficial effects of either a G_α_s biased agonist or β-arrestin biased antagonist.

Is there a clinical need for ET receptor biased ligands? Evidence is strongest from research in epithelial ovarian cancer demonstrating that ET-1 stimulated ET_A_-mediated β-arrestin signalling leads to activation of the oncogenic mediator NF-κB [21] and that β-arrestin-1 epigenetically regulates ET-1-induced β-catenin signalling [22], [23] both contributing to tumour cell proliferation, invasion and metastasis. A β-arrestin biased ET_A_ antagonist would be predicted to have benefit over a non-biased ET_A_ antagonist in ovarian cancer as ET_A_/Gαs/cAMP activation of protein kinase A opposes the detrimental ET_A_/β-arrestin stimulated expression of cancer genes. ET_A_/Gq signalling is also oncogenic therefore an alternative strategy that may demonstrate even greater target refinement would be to develop an ET_A_/Gαs biased agonist as proposed by Teoh and colleagues [24] (Fig. 2). Interestingly, pepducins have been designed for the β_2_-adrenoreceptor that selectively promote a Gαs biased conformation [25] therefore this may be one strategy that can be applied to biased targeting of the ET_A_ receptor in cancer.

Could selective activation or inhibition of ET signalling pathways in heart failure result in clinically efficacious drugs and explain the lack of benefit of endothelin receptor antagonists in heart failure clinical trials to date, despite promising evidence from pre-clinical studies? For the angiotensin-II system it has been demonstrated that β-arrestin mediated signalling in heart failure is beneficial and selective activation of this pathway using TRV027 (and thus inhibition of G-protein signalling) promotes both vasodilatation and improved cardiac function at least in animal models [26]. This compound is currently being investigated in a Phase IIb study in patients hospitalised for acute decompensated heart failure (ClinialTrials.gov identifier NCT01966601) with estimated completion March 2016. Conversely, β-arrestin signalling in cardiac fibroblasts has been proposed to contribute to detrimental ventricular remodelling [27]. It is not known if ET receptor mediated β-arrestin signalling is protective in heart failure, but if so it could be inferred that currently available endothelin antagonists that block G-protein and β-arrestin signalling or bosentan that may be a β-arrestin biased antagonist would not produce clinical benefit and may even be detrimental. However, the lack of efficacy in heart failure trials was predominantly owing to the development of peripheral oedema, thought to be a result of effects on endothelin mediated renal salt and water homeostasis rather than a lack of beneficial effect on haemodynamics [28]. It has also been suggested that the contribution of increased ET-1 to pathological cardiac remodelling in heart failure may be a result of ET_A_ mediated inhibition of reuptake of noradrenaline released from cardiac sympathetic nerves [29]. Consequently endothelin antagonists would not confer additional advantage in patients already taking β-blockers enrolled in these trials. Whether the beneficial and detrimental actions of endothelin antagonists in heart failure or other conditions such as hypertension could be discerned by the development of ligands with a particular signalling profile remains mere speculation at this time but should be investigated as the field matures.

In summary, compared to the development and exploitation of biased ligands for other GPCRs the identification of compounds that selectively engage or block a subset of ET receptor activated signalling is only now beginning to be explored. However, the possibility that the endogenous ET peptides and currently available agonists and antagonists may show pathway bias should be considered and investigated by all those with an interest in the role of the endothelin system in health and disease.

## Figures and Tables

**Table 1:** Bias analysis for the relative effectiveness of endothelin peptides in the human saphenous vein constrictor and ET_A_-mediated β-arrestin recruitment assays.

	Saphenous vein constrictor assay			β-Arrestin recruitment assay			Saphenous vein vs β-arrestin	
LogR	ΔLogR	RE	LogR	ΔLogR	RE	ΔΔLogR	Bias factor
ET-1	8.55 ± 0.15	0 ± 0.22	1	9.46 ± 0.08	0 ± 0.11	1	0 ± 0.24	1
ET-2	8.26 ± 0.21	− 0.29 ± 0.26	0.51	8.67 ± 0.03	− 0.79 ± 0.08	0.16	0.49 ± 0.27	3.1
ET-3	6.80 ± 0.37	− 1.75 ± 0.40	0.018	7.11 ± 0.03	− 2.35 ± 0.08	0.0045	0.60 ± 0.41	4.0
S6b	8.11 ± 0.12	− 0.44 ± 0.12	0.36	8.68 ± 0.06	− 0.78 ± 0.10	0.17	0.34 ± 0.15	2.2

R = (τ / K_A_), the transducer coefficient, where τ is an index of agonist efficacy and K_A_ is functional affinity of the agonist. ΔLogR is the relative LogR values of test agonists compared to the reference agonist in a particular assay. ΔΔLogR is the relative ΔLogR values for particular agonists between assays. The bias factor is determined as 10^ΔΔLogR^. Analysis performed as described by van der Westhuizen and colleagues [11].
